# 2D–3D reconstruction of distal forearm bone from actual X-ray images of the wrist using convolutional neural networks

**DOI:** 10.1038/s41598-021-94634-2

**Published:** 2021-07-27

**Authors:** Ryoya Shiode, Mototaka Kabashima, Yuta Hiasa, Kunihiro Oka, Tsuyoshi Murase, Yoshinobu Sato, Yoshito Otake

**Affiliations:** 1 Department of Orthopaedic Surgery, Osaka University Graduate School of Medicine, 2-2 Yamadaoka, Suita, Osaka 565-0871 Japan; 2 Division of Information Science, Nara Institute of Science and Technology, 8916-5 Takayama, Ikoma, Nara 630-0192 Japan

**Keywords:** Medical research, Engineering

## Abstract

The purpose of the study was to develop a deep learning network for estimating and constructing highly accurate 3D bone models directly from actual X-ray images and to verify its accuracy. The data used were 173 computed tomography (CT) images and 105 actual X-ray images of a healthy wrist joint. To compensate for the small size of the dataset, digitally reconstructed radiography (DRR) images generated from CT were used as training data instead of actual X-ray images. The DRR-like images were generated from actual X-ray images in the test and adapted to the network, and high-accuracy estimation of a 3D bone model from a small data set was possible. The 3D shape of the radius and ulna were estimated from actual X-ray images with accuracies of 1.05 ± 0.36 and 1.45 ± 0.41 mm, respectively.

## Introduction

As an imaging examination method, computed tomography (CT) is essential for (1) diagnosis, (2) pathology understanding, and (3) operation planning in orthopedics. The 3D bone models generated from CT data (CT bone models) are widely used in orthopedics for (1) visually inspecting bone morphology and joint structure and (2) quantitatively evaluating various pathological conditions. For example of (3), CT-based navigation systems have been established as an effective mean of surgical support using CT bone models^1^. Also useful for surgical support are patient-matched instruments designed using CT bone models^2–4^. In joint kinematic research, the 3D motion analysis of a living body is performed using the 2D–3D registration method, which superimposes a CT bone model on a 2D fluoroscopic image^5,6^. Thus, CT is useful as it provides a large amount of information and can be applied to image analysis, albeit at the expense of higher radiation exposure and medical costs^7^.

To address this issue, 2D–3D reconstruction has been studied, in which a 3D bone shape is estimated and constructed from 2D X-ray images without CT. Methods for estimating the 3D bone shape underlying X-ray images have been reported in which the 2D projections of the 3D statistical shape model (SSM)^8–12^ or generic model^13^ calculated from the training data set were compared with X-ray images, resulting in a similarity score, and the shape parameters of SSM or generic model were optimized to maximize the similarity. Traditionally, because most of these methods require an initial registration via manual operation, they have limitations in terms of complexity and reproducibility.

Recent works on 2D–3D reconstruction used convolutional neural networks (CNNs) to extract anatomical landmarks^14,15^. The low computational cost in the inference step in CNNs is a significant benefit in routine clinical applications. However, the previous studies^14,15^ used only CNNs for initial SSM registration. Meanwhile, in the field of computer vision, methods for directly estimating and constructing 3D shapes solely from 2D images using CNNs are being developed^16–19^. For medical images, there are no peer reviewed reports on validated 2D–3D reconstruction of the living human bones from actual X-ray images using CNNs. The novelty of the present work is the development of a CNN-based approach for 2D–3D reconstruction of living human bones, which is applicable to actual X-ray images, and the evaluation of its accuracy using a sufficient number of cases. The main difficulties in processing actual X-ray images of living human bones are (1) variation in image characteristics and noise modeling, (2) overlap of multiple bones and other surrounding tissues, and (3) construction of a sufficient quantity of training data with which actual X-ray images are associated. In this study, an approach to overcome these difficulties was developed.

Herein, we consider the feasibility of constructing a 3D bone model directly from actual X-ray images in diagnosis and surgical planning in orthopedics by introducing CNNs. Generally, when using a CNN, a large amount of data is required for training. However, it is difficult to prepare a large amount of paired training data of actual X-ray images and their corresponding CT images. Therefore, by combining (1) a method that uses digitally reconstructed radiography (DRR) images (i.e., virtual X-ray images) generated from CT data instead of actual X-ray images as training data and (2) a method that translates actual X-ray images into DRR-like images, a system that can efficiently train a CNN even using a relatively small number of actual image data sets was devised. Using this method, a 2D–3D reconstruction method that can estimate and construct the 3D bone model of the distal forearm using only the actual X-ray images of the wrist was developed. Extensive experiments using an actual clinical data set were also conducted to evaluate its applicability in routine clinical practice.

## Materials and methods

This study was approved by the institutional review board of Osaka University Hospital (approval No. 15426), which waived the need to obtain informed consent from each participant. All the followed procedures were in accordance with the ethical standards of the responsible committees on human experimentation (institutional and national) and with the 1975 Declaration of Helsinki, as revised in 2000.

### Materials

At two centers, 173 bilateral CT images of one-sided fractured or deformed wrists of 126 adults were obtained. 46 adults underwent CT scans in multiple forearm rotation positions to investigate the 3D dynamics. The CT data showed a slice thickness of 0.625 or 1.25 mm. Meanwhile, one direction of actual X-ray images of the unaffected wrists of 105 of the 126 adults who had CT images taken, excluding those with missing data, were obtained. The CT data with 1.25-mm slice thickness were interpolated into 0.625-mm intervals. The CT image size was 400 × 400 × 400 pixels for a voxel size of 0.625 × 0.625 × 0.625 mm. The X-ray image size was 500 × 625 pixels with a pixel size of 0.4 × 0.4 mm.

### Methods

#### Overview

**Figure 1:**
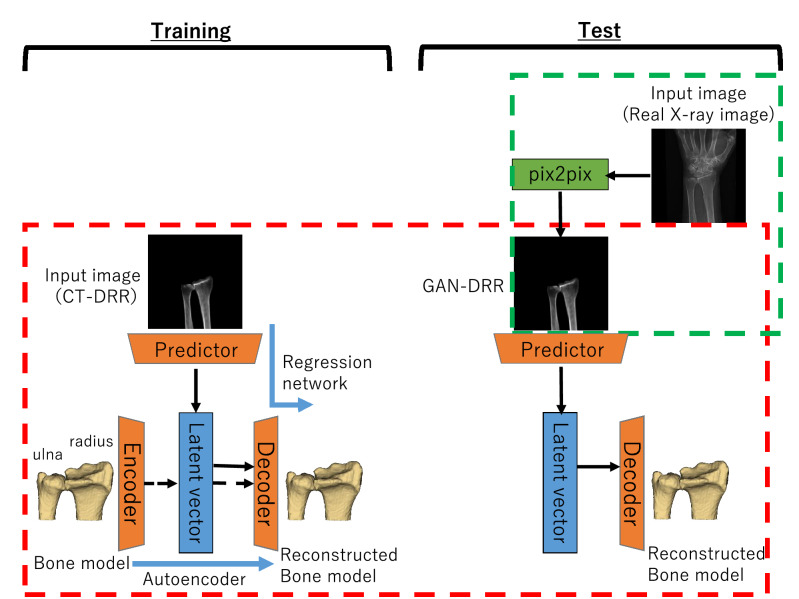
Flow chart of the proposed 2D–3D reconstruction method. The left half denotes training; the right half denotes test. Red frame: CNNs (TL net). Green frame: GAN (pix2pix).

Figure 1 shows an overview of our proposed method. The 3D bone model was estimated and constructed from actual X-ray images in two steps.

1. A generative adversarial network (GAN)^20^ inferring a DRR-like (called “GAN-DRR” in the figure) image of segmented bone from actual X-ray images (green frame in the figure).
2. CNNs inferring 3D bone reconstruction from DRR-like images (red frame in the figure).

In step (2), as the CNNs for 2D–3D reconstruction, the TL-embedding network proposed by Girdhar et al*.*^18^ (hereinafter referred to as TL net) was used, which included a decoder that was pretrained using an autoencoder and achieved high-precision 2D–3D reconstruction. To prepare a data set that was sufficiently large to train the TL net, data augmentation was performed using DRR images created by arbitrarily rotating CT data and projecting onto a 2D plane. The advantage of using DRR images for training was that a large quantity of images could be automatically generated from a CT image, allowing effective learning. The GAN reported by Goodfellow et al*.*^20^ generated images from random variables through adversarial training. Many GAN variants have previously been proposed, among which one variant specializing in image translation reportedly obtained clear images with higher resolution than those obtained from an image translation using a conventional CNN^21,22^. A type of GAN called pix2pix, proposed by Isora et al. was used, which enabled high-precision image translation through supervised learning^23^. The following section explains the way to construct a data set and train pix2pix in stages.

#### Image translation from actual X-ray image to DRR-like image using pix2pix

##### Data set

**Figure 2:**
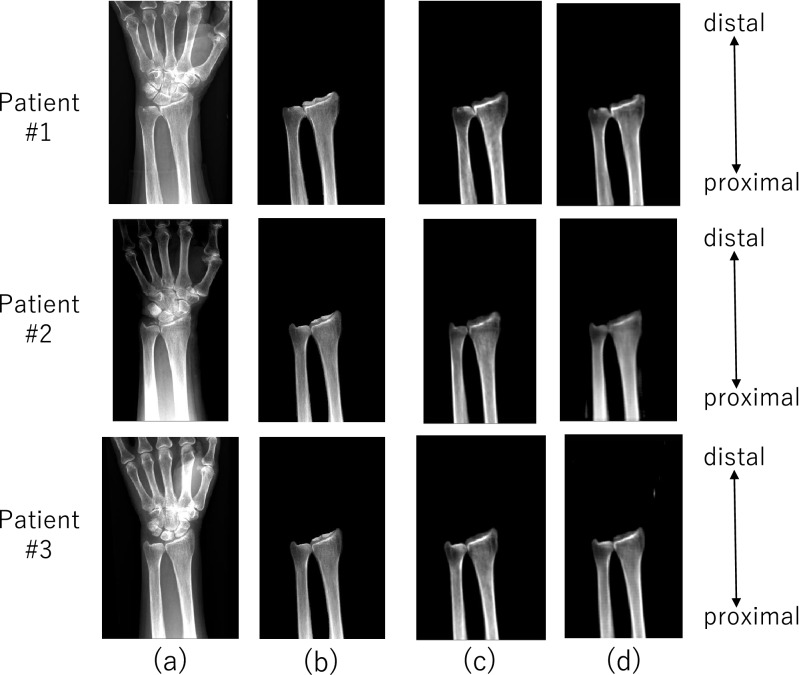
Image translation: (**a**) actual X-ray image; (**b**) actual X-ray image showing segmented radius and ulna; (**c**) registration-DRR; (d) GAN-DRR.

To construct a training data set for pix2pix, 33 pairs of actual X-ray (Fig. 2a) and CT images (i.e., 30% of the data) randomly selected from 105 pairs were used. Using these data, a data set was constructed as follows.

1. The radius and ulna on the X-ray (Fig. 2b) and CT images were manually segmented to extract 3D labels. The extraction of the region of interest allowed the objective anatomical features of the radius and ulna to be recognized with high accuracy.
2. DRR images were automatically generated from CT images aligned to actual X-ray images using the intensity-based 2D–3D registration method^24,25^ that utilized cross correlation of the intensity gradients as the similarity metric; this DRR was called “registration-DRR” (Fig. 2c).
3. The data were augmented 100 times by random 2D affine transformation (translation, rotation, scaling and shear) of each actual X-ray image and registration-DRR images to generate 3300 pairs of data. The validity of the augmented data was visually verified by an expert surgeon.

##### Training

pix2pix was trained using 3,300 pairs of actual X-ray images and their corresponding registration-DRR images. A DRR-like image translated from an actual X-ray image by pix2pix (a type of GAN) after training is denoted as GAN-DRR (Fig. 2d).

#### Estimation and construction of 3D image from 2D images using CNNs (TL net)

##### Data set

**Figure 3:**
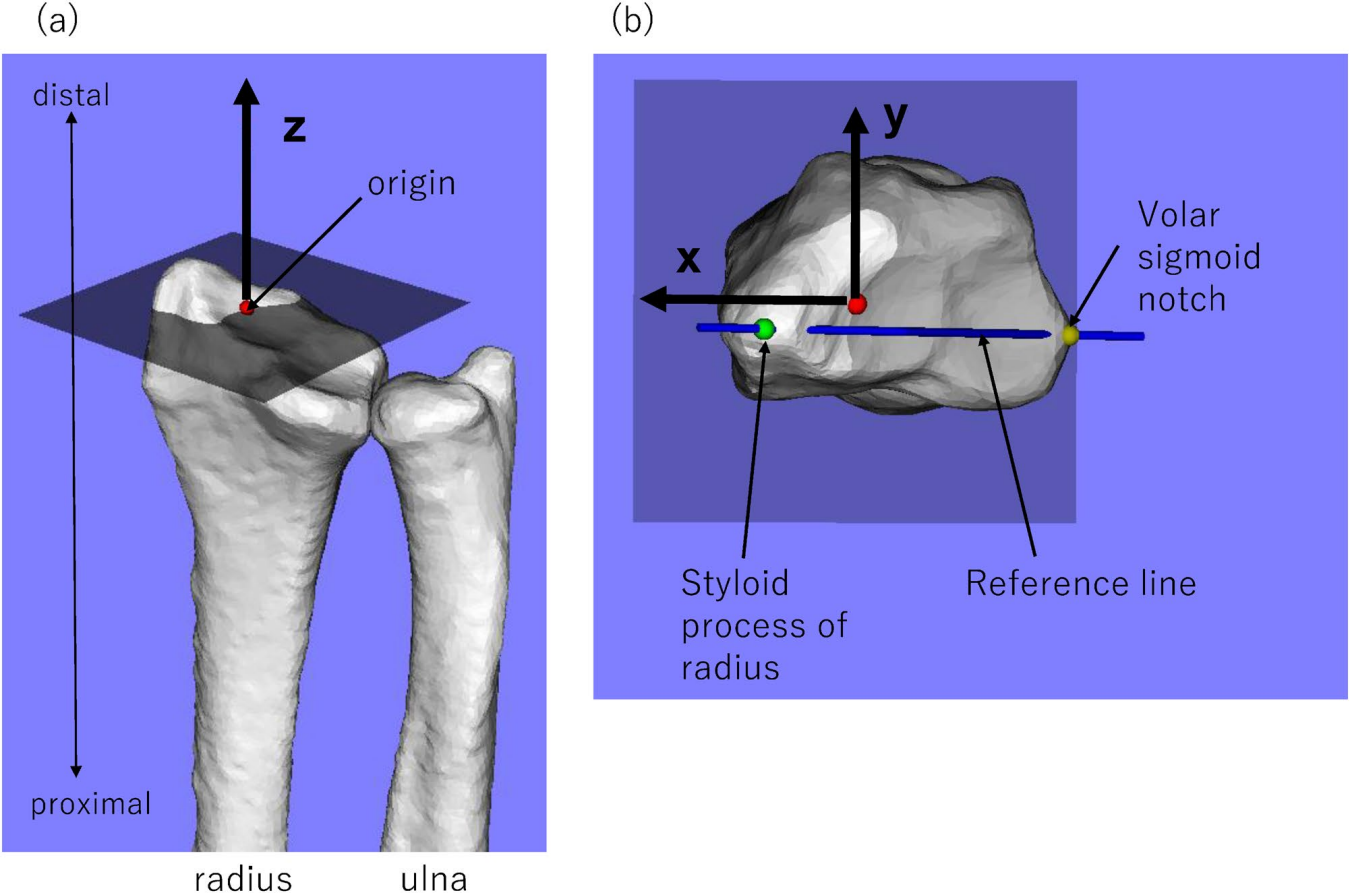
The relationship between image translation accuracy and 2D–3D reconstruction accuracy.

When constructing the data set used for training TL net, the CT images were geometrically normalized as follows. The line connecting the styloid process of the radius and the volar sigmoid notch of the distal radioulnar joint was used as the reference line, the radial axis was used as the Z axis and the intersection point between the Z axis and the distal articular surface of the radius was used as the origin (Fig. 3a). On a plane passing through the origin and perpendicular to the Z axis, a line passing through the origin parallel to the line projected from the reference line was set as the X axis and a line passing through the origin and perpendicular to the X axis was set as the Y axis (Fig. 3b). The direction of rotation of the radius around the Z axis was unified without changing the relative position of the radius and ulna. The field of view uses a range of ± 40 mm in the Z-axis direction from the origin. Consequently, the estimated object in this study became the shape of the radius and ulna in the range of ± 40 mm from the origin. ‘CT-DRR’ (cf., other DRRs used in this paper: GAN-DRR, registration-DRR) was defined as DRR generated from this geometrically normalized CT images. In generating the CT-DRR, the CT images were translated and randomly rotated in a range of ± 30°, ± 30 mm along the X, Y, and Z axes to augment the data 40 times to 6920 CT volumes.

##### Training

The flow of 2D–3D reconstruction using the TL net is shown in Fig. 1. The TL net learned in the following three steps.

1. The encoder and decoder were trained using an autoencoder with 3D labels of distal radius and ulna as the input and output. Overfitting of the autoencoder was suppressed by providing Gaussian noise to the input image. This process is generally understood to change the training process toward suppression of overfitting as proposed in the denoising autoencoder^26^.
2. The predictor for extracting a latent vector from CT-DRR was trained to reconstruct the corresponding 3D labels of the distal radius and ulna by concatenating the decoder learned in (1). The parameters in the decoder were fixed.
3. For fine tuning, the predictor and decoder were trained using CT-DRR and 3D label as the input and the 3D label as the output while fixing the encoder parameters. This step was slightly modified from the original paper^18^, as we experimentally found better training stability by fixing the encoder parameters but found no accuracy improvement when they were unfixed. The training was performed with loss terms representing the fitness of (a) the latent vector extracted from the encoder and predictor and (b) the reconstructed 3D label.

In the above training, optimizations were performed by minimizing the following respective loss functions:

$$ \begin{aligned} L_{step1} & = L_{label} \left( {D\left( {E\left( {y_{noised} } \right)} \right),y} \right)\lambda_{1} \left| {E\left( {y_{noised} } \right)} \right|_{1} , \\ L_{step2} & = L_{label} \left( {D\left( {P\left( x \right)} \right),y} \right){ + }\lambda_{1} \left| {P\left( x \right)} \right|_{1} , \\ L_{step3} & = L_{label} \left( {D\left( {P\left( x \right)} \right),y} \right){ + }\lambda_{1} \left| {P\left( x \right)} \right|_{1} + \lambda_{2} \left| {E\left( y \right) - E\left( {D\left( {P\left( x \right)} \right)} \right)} \right|_{2} , \\ \end{aligned} $$

where *E*, *P*, and *D* indicate the output of the encoder, decoder, and predictor, respectively, and *x*, *y,* and $y_{noised}$ indicated a 2D image (CT-DRR), 3D label, and 3D label with Gaussian noise, respectively. $\lambda_{1}$ and $\lambda_{2}$ were hyper parameters, both of which were $10^{ - 4}$ in this study. The function $L_{label} \left( { \cdot , \cdot } \right)$ was the softmax cross entropy. At all stages, L1 normalization was performed to suppress the complexity of the latent variables obtained from the encoder or predictor.

Using the error functions described above, the network was updated 35,000 times in (1) and (2) and 15 000 times in (3). Adam’s method^27^ was used to optimize the parameters at all stages. The learning rates at each stage were 10^−4^, 10^−4^, and 10^−5^, respectively.

### Experiments

#### Experiment 1: accuracy evaluation of image translation from actual X-ray image to DRR-like image using pix2pix

The accuracy of the image translation from actual X-ray images to GAN-DRR (Fig. 2d) was quantitatively assessed. The registration-DRR was used as the ground truth, and the intensity difference by GAN-DRR was quantified using the mean absolute error (MAE). The experiment validated a dataset of 33 patients with four-fold cross validation (three-fourth patients for training data, one-fourth patients for test data). Of the 100-fold expanded training data, 85% were used for training and 15% were used for validation.

#### Experiment 2: accuracy evaluation of 2D–3D reconstruction using TL net

To verify the accuracy of the 2D–3D reconstruction using the TL net, two types of experiment were performed, i.e., accuracy evaluation of the (1) generation of a 3D bone model from CT-DRR and (2) generation of a 3D bone model using GAN-DRR translated from an actual X-ray image. The TL net trained using CT-DRR was used in both cases. In experiment (1), 173 images of 126 patients were validated; in experiment (2), 72 images of 72 patients, excluding 33 patients used for training pix2pix out of 105 patients, were validated with four-fold cross validation (three-fourth patients for training data, one-fourth patients for test data). Of the 40-fold expanded training data, 85% were used for training and 15% were used for validation.

For the evaluation in each experiment, the ground truths were those of a CT bone model and the error between the CT bone model and the model generated in each experiment was evaluated using the average symmetric surface distance (ASD) expressed by

$$ ASD = \frac{1}{{\left| {S_{A} } \right| + \left| {S_{B} } \right|}}\left( {\mathop \sum \limits_{{p_{A} \in {\text{S}}_{A} }} \mathop {\min }\limits_{{p_{B} \in S_{B} }} d\left( {p_{A} ,p_{B} } \right) + \mathop \sum \limits_{{p_{B} \in {\text{S}}_{B} }} \mathop {\min }\limits_{{p_{A} \in S_{A} }} d\left( {p_{A} ,p_{B} } \right)} \right) $$

where *d* was the Euclidean distance between the two points and $S_{A}$ and $S_{B}$ were the point cloud models of ground truth and reconstruction results. The point cloud model was generated from the volume by the Marching cubes method.

#### Experiment 3: experiments to study sensitivity of the image translation accuracy on the 2D–3D reconstruction accuracy

To examine how the image translation accuracy of pix2pix affects the 2D–3D reconstruction accuracy, in this experiment, the following four types of 2D image were input to the TL net that was trained using CT-DRR to reconstruct a 3D bone model (2D–3D reconstruction) and the error with the CT bone model (ground truth) was compared using ASD:

1. CT-DRR (DRR generated by CT without using the actual X-ray image, which was used in the training);
2. GAN-DRR (DRR-like image generated from actual X-ray image using pix2pix);
3. registration-DRR (DRR generated by 2D–3D registration of CT and actual X-ray images);
4. bone-segmented, histogram-matched, actual X-ray images: actual X-ray images using simple image processing based on histogram matching^28^ using registration-DRR as a template and segmentation of radioulnar bone.

GAN-DRR was proposed, which led to a corresponding 2D–3D reconstruction result.

**Figure 4:**
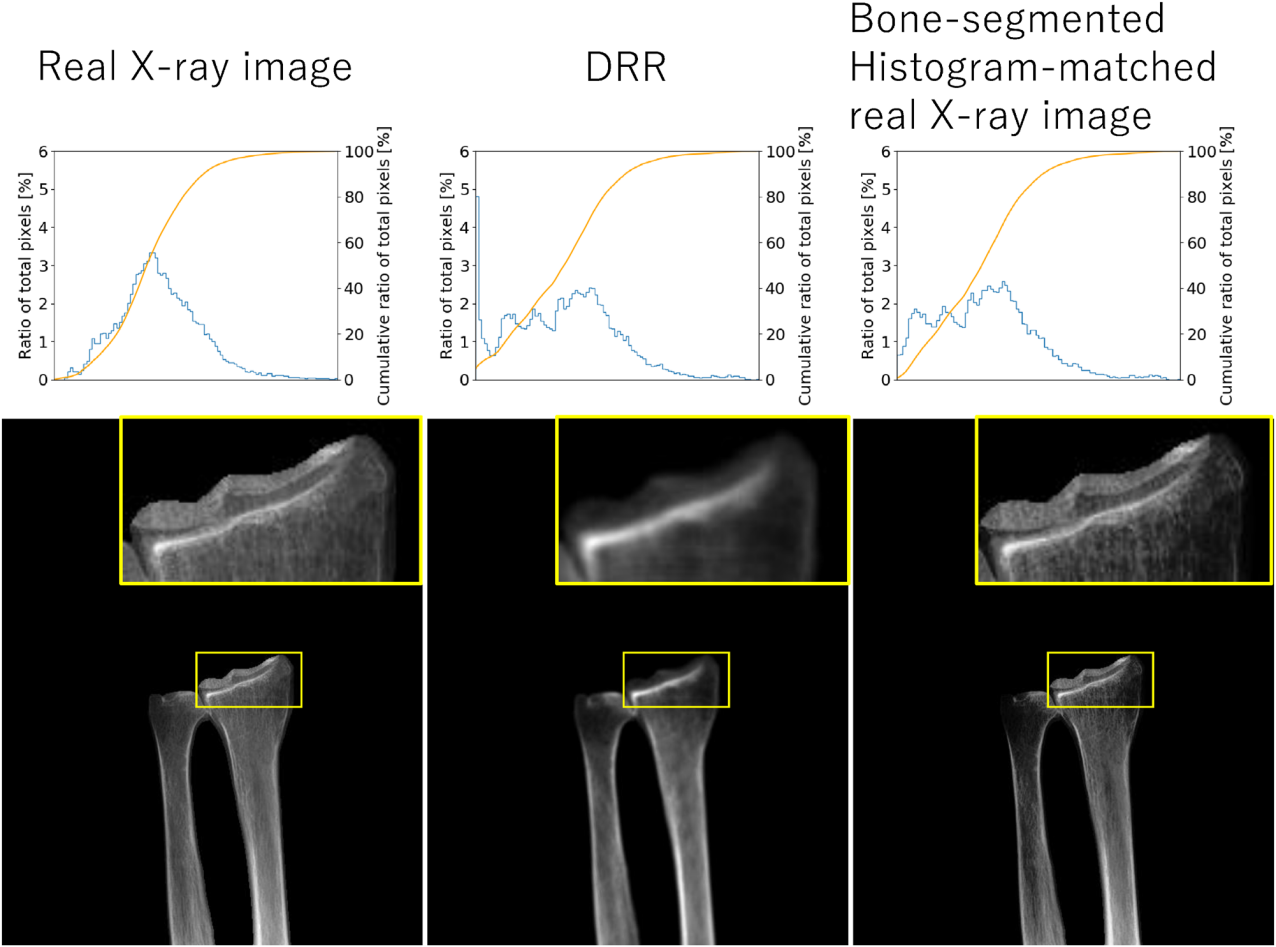
Visualization result of mean absolute error (MAE): (**a**) example of small error, MAE: 0.016 ± 0.020; (**b**) example of large error, MAE: 0.051 ± 0.027.

The bone-segmented, histogram-matched, actual X-ray image was generated to obtain baseline data that did not use GAN-DRR. A potential approach to generate a DRR-like image from an actual X-ray image is to simply perform segmentation of the target bone. To obtain better contrast that yields improved accuracy in 2D–3D reconstruction, histogram-equalization was applied to the bone-segmented image. (Fig. 4).

**Figure 5:**
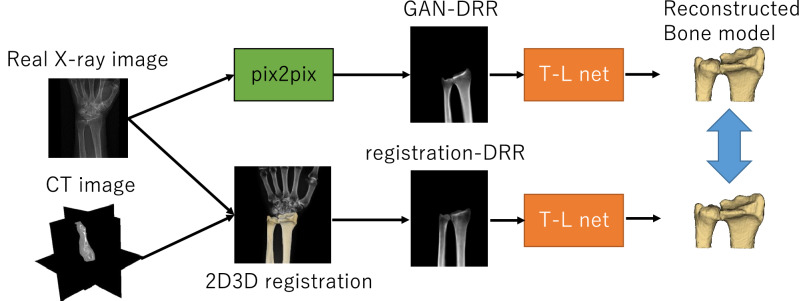
Adaptive results of a bone-segmented, histogram-matched, actual X-ray image. Blue line: ratio of total pixels. Orange line: cumulative ratio of total pixels.

As registration-DRR was used for training pix2pix, it was considered to be a truth for image translation. Therefore, if the difference between the error in the 2D–3D reconstruction by GAN-DRR and registration-DRR was small, the image translation using pix2pix was considered accurate (Fig. 5).

### Statistical analyses

All statistical analyses were performed using the JMP Pro 14 software (SAS, USA). The Shapiro–Wilk test was used to determine the normality of the data distribution. The data are presented as averages and standard deviations. The differences in accuracy of the 2D–3D reconstruction results among the four types of 2D image were evaluated using the Tukey–Kramer HSD test. *p* values < 0.05 were considered statistically significant.

## Results

### Experiment 1

**Figure 6:**
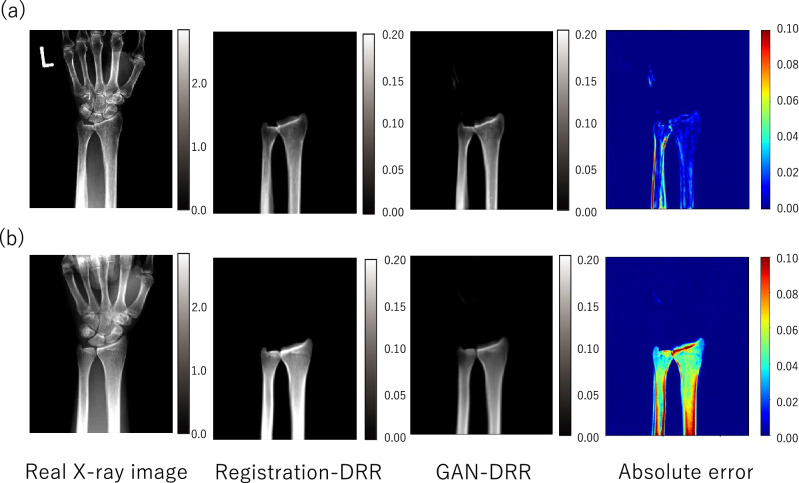
Mean absolute error (MAE) of image translation for each patient.

The results of the image translation from the actual X-ray images to DRR-like images by pix2pix are shown in Fig. 2d. GAN-DRR generated from actual radiographs retained the distal radioulnar anatomy. Two example results with their MAEs are depicted in Fig. 6. The DRRs in the training dataset were generated by considering the line integral of the linear attenuation coefficient derived from the CT value (in Hounsfield units), resulting in the approximate range of [0.00–0.20] as shown for registration-DRR. The MAE of GAN-DRR using registration-DRR as the ground truth was 0.016 ± 0.020 for case A and 0.051 ± 0.027 for case B. The total MAE was 0.025 ± 0.020, which suggests an error magnitude of approximately one-tenth of the intensity range.

### Experiments 2 and 3

**Figure 7:**
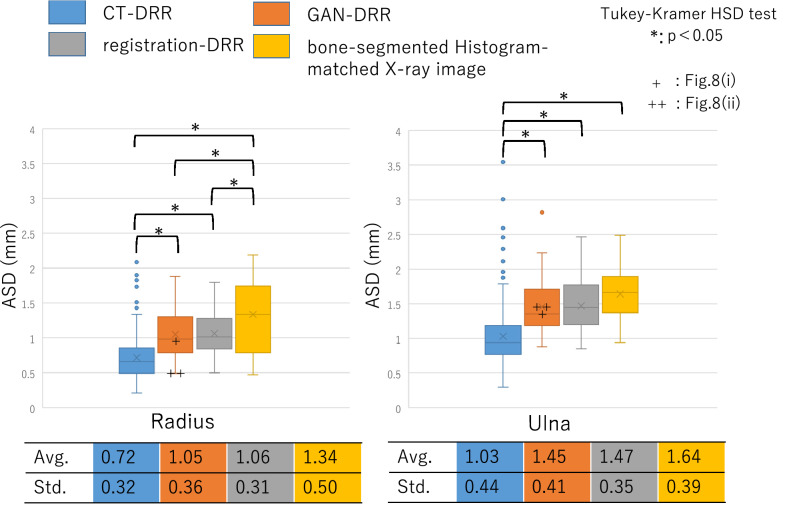
Experiment evaluating the relationship between image translation accuracy and 2D–3D reconstruction accuracy.

**Figure 8:**
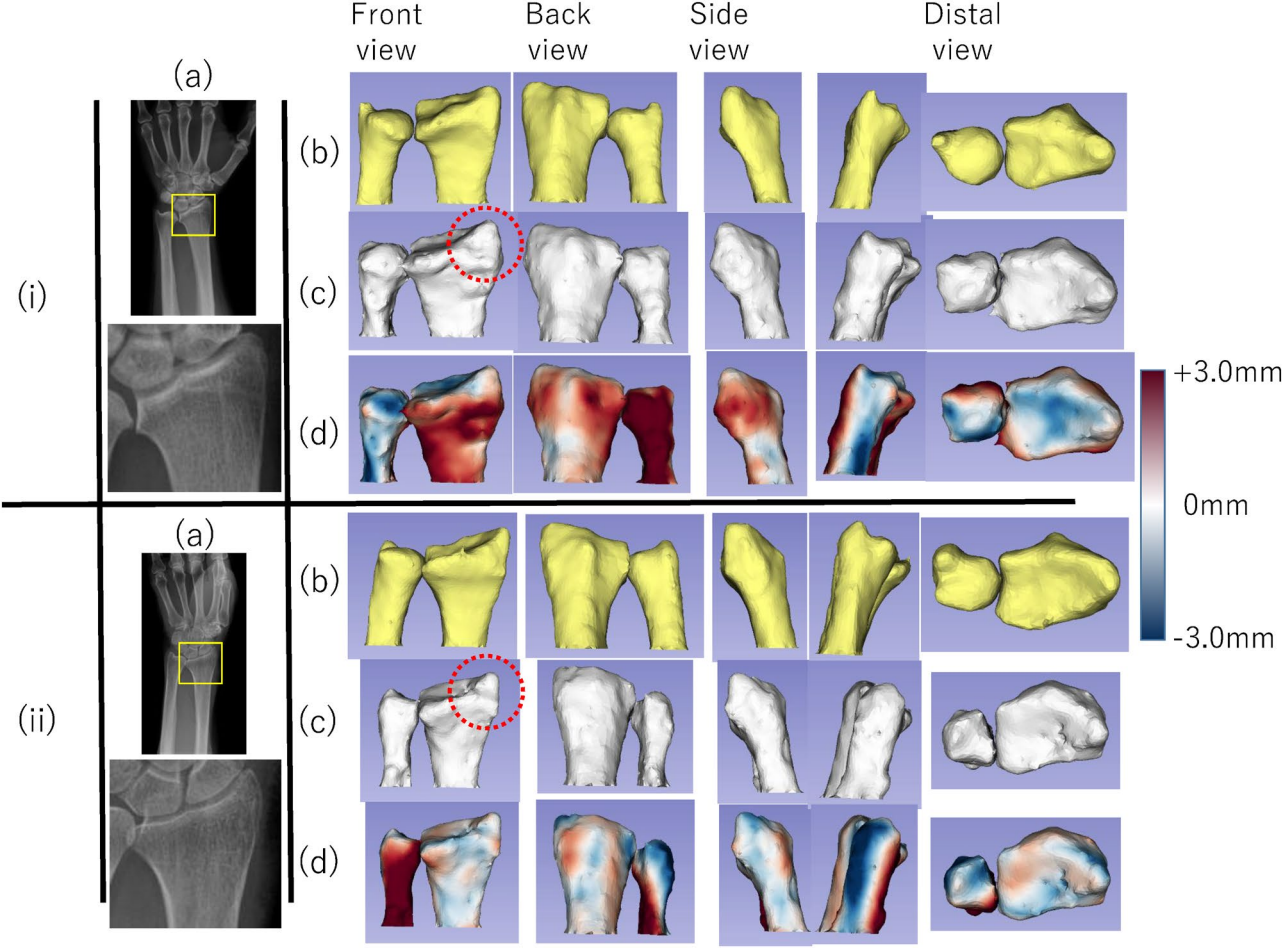
Example of the 2D–3D reconstruction result for actual X-ray images. (i) Average result (ASDs: radius, 0.95 mm; ulna, 1.34 mm). (**a**) actual X-ray image (**b**) ground truth; (**c**) 3D bone model estimated by GAN-DRR; (d) visualization of ASD. (**ii**) Good result (ASDs: radius, 0.49 mm; ulna, 1.50 mm). (**a**) actual X-ray image (**b**) ground truth; (**c**) 3D bone model estimated by GAN-DRR; (**d**) visualization of ASD. Red circle in (**c**) showed that the shape of the radial styloid process was reconstructed in each case.

Figure 7 shows the accuracies of the 2D–3D reconstruction of the four types of 2D images. In the experiments using CT-DRR, the estimated accuracies of the 2D–3D reconstruction results for the radius and ulna were 0.72 ± 0.32 and 1.03 ± 0.44 mm, respectively. In the experiments using GAN-DRR, the estimated accuracies of the 2D–3D reconstruction results for the radius and ulna were 1.05 ± 0.36 and 1.45 ± 0.41 mm, respectively. The 3D bone models of the two cases estimated from GAN-DRR are shown in Fig. 8. In the radius, the accuracies of the 2D–3D reconstruction results estimated from CT-DRR and GAN-DRR were both < 1.25 mm, which is the resolution of the CT. In the ulna, the accuracy of the 2D–3D reconstruction result estimated from CT-DRR was < 1.25 mm, but that estimated from GAN-DRR was > 1.25 mm. In each bone, the accuracy of the 2D–3D reconstruction result differed significantly between CT-DRR and GAN-DRR.

In the experiments using registration-DRR, the estimated accuracies of the 2D–3D reconstruction results for the radius and ulna were 1.06 ± 0.31 and 1.47 ± 0.35 mm, respectively. In each bone, the accuracy of the 2D–3D reconstruction result differed significantly between CT-DRR and registration-DRR, while no significant difference was observed between GAN-DRR and registration-DRR. In the experiments using bone-segmented histogram-matched actual X-ray images, the estimated accuracies of the 2D–3D reconstruction results for the radius and ulna were 1.34 ± 0.50 and 1.64 ± 0.39 mm, respectively. The error of each 3D bone model was significantly smaller than that estimated from bone-segmented histogram-matched actual X-ray images (*p* < 0.05 for CT-DRR versus bone-segmented histogram-matched actual X-ray images; *p* < 0.05 for GAN-DRR versus a bone-segmented histogram-matched actual X-ray image; *p* < 0.05 for registration-DRR versus a bone-segmented histogram-matched actual X-ray image).

## Discussion

### Problems arising while using CNNs for medical images and the solutions in this study

While CT bone models are used widely in clinical settings because of their convenience and usefulness, radiation exposure and medical costs pose problems. To solve these problems, a method was developed for estimating 3D bone models directly from only actual X-ray images using CNNs. However, a large database is generally required to train CNNs, and it is not easy to prepare a large amount of paired data of actual X-ray images and CT images as in this study. To address this limitation, DRR images generated from CT were used, which were called CT-DRR, to augment the data. The advantages of using DRR images are as follows: As CT-DRR data is generated from CT data, it is possible to generate an arbitrary number of images in any imaging direction. Therefore, a large number of data sets that reproduce variations such as differences in photographic position and angle can be constructed. This makes it possible to apply GAN-DRR generated from actual X-ray images using limited data to the TL net trained sufficiently by CT-DRR. Another advantage is that the effects of various imaging parameters related to the contrast of the image, such as X-ray irradiation time and irradiation dose, can be removed from the input image because the intensity of CT in Hounsfield units is normalized so that it linearly correlates with density. The final advantage is that the DRR created from bone-only CT data (i.e., CT volume for the case of extracted bones) supresses the noise caused by the surrounding soft tissue.

### Previous studies of 2D–3D reconstruction using an SSM or generic model in medical images

Previous research has estimated 3D bone models from X-ray images^8–13^. The methods in these studies involved superimposing an SSM or generic model on X-ray images and deforming it into the optimal 3D shape. In proximal and distal femurs, high-accuracy estimates have been reported, with an intersurface error of 0.68–1.0 mm^8–11^. Most of the conventional SSM-based 2D–3D reconstruction methods incur high computational costs and are operationally complicated, including manual operation during the initial registration. Some methods do not require manual operation, but the accuracy of reconstruction is inferior (3.04 mm)^12^, and the accuracy of reconstruction has not been evaluated^13^. There are some reports of SSM-based 2D–3D reconstruction methods using CNNs^14,15^. Although these methods use CNNs only for initial SSM registration to detect anatomical landmarks and facilitate 3D modeling, they still incur high computational costs.

### Previous studies of 2D–3D reconstruction using CNNs in other fields

Meanwhile, in the field of computer vision, recent studies have estimated 3D models directly from 2D images using CNNs^16–19^. These studies have estimated (1) 3D models from 2D images of furniture and airplanes and (2) 3D bone models from X-ray images of animal bones. Since these methods do not require registration, they are generally faster than methods involving registration.

### Differences from a study of 2D–3D reconstruction using CNNs in medical images

In medical imaging, the use of CNNs for 2D–3D reconstruction of human knee bones directly from actual X-ray images continues to be reported^29^. These studies reconstructed the four bones of the knee (femur, patella, tibia and fibula) with an average chamfer of 1.87 mm. As in our method, they used DRR images generated from CT data as training data, translated into DRR-like images when adapting the X-ray images. They used a simple network with only an encoder and a decoder as a network for 2D–3D reconstruction, with Cycle GAN^30^ for image translation. On the other hand, they used biplanar X-ray images as 2D images, even though biplanar X-ray images are not commonly used clinically. In the present study, 2D–3D reconstruction was accomplished from images obtained using a single X-ray source, with wide clinical relevance. As single-source X-ray images were used, an advanced network (TL net) was introduced for 2D–3D reconstruction that required strong preprocessing and a network (pix2pix) that was trained on paired datasets for image translation.

### Advantages of the network used in this study

The TL net network used in the present study was used for segmentation by Oktay et al*.*^31^, who reported that the anatomical features of the living heart could be successfully learned. In contrast to the image translation proposed by Kaste et al*.*^29^, trained by an “unpaired” dataset (i.e., nonalignment of X-ray image and DRR), a “paired” dataset using 2D–3D registration^24^^,^^25^ was generated and pix2pix^23^ was applied, allowing more efficient training with a smaller dataset compared with unpaired training. Image translation from actual X-ray images using GAN has previously been applied to chest radiographs^32,33^ and the spine^34^. However, conventional methods reportedly limited by partial inconsistencies in translations of anatomical features between different modalities^35^. In the present study, it was attempted to address the problem using DRR derived from segmented CT, such that the image consisted solely of the region of interest, which potentially contributes to improved image translation accuracy.

### Accuracy of image translation using pix2pix in this study

In 2D–3D reconstruction from biplanar X-ray images, contour information can be effectively used, whereas in 2D–3D reconstruction from single-direction X-ray images, intensity information is important. In this study, image translation that restored the intensity information from actual X-ray images was performed and showed that the accuracy of image translation contributed to the accuracy of 2D–3D reconstruction. The accuracy of image translation from actual X-ray images to DRR-like images by pix2pix was confirmed to be as high as MAE = 0.025 ± 0.020, and the reconstruction accuracy from the DRR-like images was also high. In the evaluation of the 2D–3D reconstruction accuracy of the radius, which is used to construct 3D models from CT-DRR and GAN-DRR, sufficient reconstruction accuracy was achieved considering the resolution (1.25 mm) of the training data (Fig. 7). Furthermore, as a result of evaluating the relationship between the image translation accuracy using pix2pix (MAE) and the 2D–3D reconstruction accuracy (ASD), GAN-DRR was able to construct a 3D image with higher accuracy than DRR-like images obtained with simple image processing on actual X-ray images based on histogram matching (Fig. 7). The accuracy of 2D–3D reconstruction estimated using GAN-DRR was also confirmed to be equivalent to that of 2D–3D reconstruction estimated using registration-DRR. This suggests a sufficient accuracy of image translation with pix2pix.

### Accuracy of 2D–3D reconstruction in this study

In this study, a high-precision 3D model was constructed from actual X-ray images with a radius of 1.05 ± 0.36 mm and an ulna of 1.45 ± 0.41 mm, with room for improvement. The estimated accuracy of the ulna was inferior to that of the radius (Fig. 8). In this study, CT images used as training data were geometrically normalized with respect to the radius, so there are variations in ulnar rotational positions. This variation is considered to be the factor that increased the estimation error of the ulna. The accuracy of the ulna can be improved by using unified data of the forearm rotational position on CT imaging or by performing 2D–3D reconstruction using actual X-ray images in two directions.

### Limitations in this study

The first limitation of this study is that only the healthy distal forearm was involved. The study initially considered only distal forearm considering the increasing number of patients with wrist fracture^36^; further, it was thought that it would be useful to estimate the shape of the healthy bone to be used as a reduction target when planning fracture treatment or simulating operation for deformed cases. The adaptation of this method for cases of disease and other bones should be investigated in the future. The second limitation is that the data were limited. This is a fundamental problem in applying medical images to CNNs. In this paper, the data were augmented using DRR to vary the simulation, and although the data before the augmentation were small, sufficient accuracy was achieved at the resolution of the current training data.

## Summary

Constructing a highly accurate 3D bone model from actual X-ray images alone with this method would likely solve the problems of (1) exposure dose in CT imaging and (2) medical cost, and the usefulness in the clinical field would be high. In the future, by adapting to the deformity of the distal forearm and other bones, high-quality medical care can be provided to children and pregnant women, who should refrain from radiation exposure as much as possible.

## Conclusions

This paper developed CNNs for estimating a 3D bone model solely based on 2D clinical X-ray images of living human bones. Extensive experiments using an actual clinical dataset were conducted to evaluate the applicability of the model in routine clinical practice. The 3D shapes of the radius and ulna were reconstructed from actual X-ray images with accuracies of 1.05 ± 0.36 and 1.45 ± 0.41 mm, respectively. To the best of our knowledge, this is the first peer reviewed report on the accuracy evaluation of CNN-based 3D reconstruction of the living human bones from clinical X-ray images.

In this study, two CNNs were used—a CNN for image translation from clinical X-ray images to DRR images and a CNN for 3D reconstruction from DRR images. The former was trained from 3,300 pairs of images augmented from 33 patient DRRs and clinical X-ray images registered with CTs, from which the DRRs were synthesized, to remove the effects of non-target tissues and noise in actual X-ray images. The latter was trained from 6,920 pairs of DRR and CT images, which were augmented by random translations and rotations of 173 CT images of 126 patients, to accommodate various positions of the bones. By combining these two CNNs, accurate 2D–3D reconstruction from clinical X-ray images has become possible, given a small number of patient data.

This study might contribute to reducing the frequency of CT scans in orthopedics. CT is useful as it provides a large amount of information and can be applied to accurate 3D reconstruction, albeit at the expense of higher radiation exposure and medical costs. Our method of 3D bone reconstruction from X-ray images incurs much less radiation exposure. Thus, it is expected to partly solve one of the problems in CT and would be potentially useful in the clinical field.
